# Plasma and bronchoalveolar lavage samples in acute lung allograft rejection: the potential role of cytokines as diagnostic markers

**DOI:** 10.1186/s12931-017-0634-6

**Published:** 2017-08-07

**Authors:** Nicole E. Speck, Macé M. Schuurmans, Christian Benden, Cécile A. Robinson, Lars C. Huber

**Affiliations:** 1 Division of Pulmonology, University Hospital Zurich, Rämistrasse 100, CH-8091 Zurich, Switzerland; 2 Clinic for Internal Medicine, City Hospital Triemli, Birmensdorferstrasse 497, CH-8063 Zurich, Switzerland

**Keywords:** Blood, Bronchoalveolar lavage, Cytokine, Diagnosis, Graft rejection, Lung transplantation, Plasma

## Abstract

The role of differential cytology patterns in peripheral blood and bronchoalveolar lavage samples is increasingly investigated as a potential adjunct to diagnose acute and chronic allograft dysfunction after lung transplantation. While these profiles might facilitate the diagnosis of acute cellular rejection, low sensitivity and specificity of these patterns limit direct translation in a clinical setting. In this context, the identification of other biomarkers is needed. This review article gives an overview of cytokine profiles of plasma and bronchoalveolar lavage samples during acute cellular rejection. The value of these cytokines in supporting the diagnosis of acute cellular rejection is discussed. Current findings on the topic are highlighted and experimental settings for future research projects are identified.

## Background

Lung transplantation is an established therapeutic option for selected patients with end-stage lung disease. While survival after lung transplantation has increased over time due to improved survival in the early post-transplant period, chronic lung allograft dysfunction (CLAD) and infections are the main factors limiting long-term survival [1] Acute cellular rejection (ACR) is a potential risk factor for the development of CLAD [2–4]. The gold standard to detect ACR is histopathological grading of transbronchial biopsies (TBB). However, these biopsies are invasive and accurate grading is limited by sampling error and interobserver variability [5–7]. Furthermore, the clinical relevance of low grade (A1) rejection is controversial and treatments vary between centres [8–10].

We have recently reviewed the role of differential cytology patterns in samples obtained from peripheral blood (PB) and bronchoalveolar lavage (BAL) for the development of ACR [11]. While these profiles show interesting trends that may facilitate early diagnosis and treatment of ACR, low sensitivity and specificity of these findings limit the clinical utility and, currently, preclude the isolated use of cellular patterns for the diagnosis of ACR. In this context, identification of other biomarkers is needed to improve the diagnostic performance of cytokine patterns for diagnosis of AR. We summarise here the experimental and clinical evidence on cytokine profiles in BAL and plasma samples during ACR, discuss limitations and outline areas for future research.

## Methods

We searched the electronic databases Medline (Bethesda, MD, USA: U.S. National Library of Medicine), EMBASE (Amsterdam, NL: Elsevier B.V.) and Web of Science Core Collection (New York, NY, USA: Thomson Reuters). Medical subject heading (MeSH) terms included “cytokines”, “bronchoalveolar lavage”, “blood plasma”, “graft rejection” and “lung transplantation”.

Publications were eligible if they provided information on cytokine patterns in BAL or PB during ACR. We considered articles published in English until 31 October 2016. This included experimental studies, prospective and retrospective clinical studies, review articles and case reports. No other restrictions were applied. We then selected those articles that fulfilled our inclusion criteria. Additionally, we scanned the references of all selected articles to find additional literature that was related to our research question. Finally, 38 papers were eligible to be included in our review. A list of the type and number of articles included is provided in Table 1.

**Table 1:** Types and number of references included in this review article

Content	Study design	Number of studies included	Number of patients	Number of samples
Cytokines	Experimental	9	-	-
	Prospective	13	407	1301 BAL 17 serum
	Retrospective	12	492	834 BAL 58 serum
	Review article	4	-	-
Total		38	899	2135 BAL 75 serum

We then evaluated the selected articles and compiled an extensive table, listing every cytokine, the reference that mentioned this parameter as well as the observed data. While writing the review article more papers were drawn on for background information. Each author reviewed the entire document and provided input before the final manuscript was completed.

### Cytokines in BAL and plasma samples

Cytokines influence inflammatory and immune reactions by mediating communication between cells. Cytokines are cell-derived non-antibody proteins, peptides or glycoproteins that activate cells in an autocrine or paracrine fashion and result in stimulatory or inhibitory effects [12]. They play a vital role in recruitment, activation, proliferation or differentiation of regulatory and effector cells of the immune system [13]. Cytokines can be divided into six groups according to their functional or structural similarities (overview provided in Table 2). While cytokines function in a complex network with a degree of redundancy, the effects of certain cytokines on specific cell types are still under investigation [12].

**Table 2:** Overview of six groups of cytokines

Group	Characteristic feature	Reference
Interleukin (IL)	Large number of cytokines named with a numeric suffix roughly in the order of discovery or molecular characterization.	[91]
Interferon (IFN)	Named for their function to interfere with viral infection, inhibiting viral replication within host cells.	[92]
Tumor necrosis factor (TNF) superfamily	Structurally homologous transmembrane proteins that typically form homotrimers.	[73]
Transforming growth factor (TGF) superfamily	Involved in both tumor development and dissemination.	[93]
Colony-stimulating factor (CSF)	Stimulate proliferation and expansion of bone marrow progenitor cells, leading to formation of erythrocytes, granulocytes, monocytes and lymphocytes.	[94]
Chemokine	Structurally homologous, low-molecular weight cytokines that promote movement and migration of all immune cells between blood and tissue.	[95]

Cytokines influence success and outcome of organ transplantation substantially [14, 15]. Allograft rejection is a multifactorial process, resulting from integration of different components of the immune system [15]. Initial activation of the innate immune system due to local tissue damage and ischemia-reperfusion injury might trigger and amplify the subsequent dysregulation of the adaptive immune response. Cytokines are produced during both phases of this process [15]. ACR has been associated with dysregulated expression of cytokines in different solid organ transplants [16–18].

Multiple ways to measure and quantify the expression of cytokines in BAL fluid and plasma samples exist, including immunohistochemistry [19], in situ hybridization [19], real-time polymerase chain reaction (RT-PCR) [20], enzyme-linked immunosorbent assays (ELISA) [21], flow cytometry (FCM) [22], bioassays [23], and other immunoassays [12]. ELISA allows detection of proteins; the concentration of the respective molecule can then be interpolated from standard curves. As such, it is the most frequently used technique [12]. Multi-parameter FCM allows quantifying many immune cell subsets and cytokine mediators with great flexibility [24]. Among novel molecular methods, microarrays permit the screening of a large number of genes, while polymerase chain reaction (PCR) is more useful to address hypotheses [25].

Many factors can potentially influence the measurements of cytokine levels, and the awareness of these factors is low [12]. Analysing cytokines in BAL fluid during rejection is further challenging since the alveolar-capillary barrier loses integrity and, subsequently, is more permeable to serum proteins during rejection [14].

Although promising results have been achieved in several areas of medical investigation, cytokines are not routinely measured in clinical practice [12, 26]. The comparability between the above-mentioned methods is unclear as the influence of various analytical and pre-analytical factors on the results are complex and still need to be elucidated [12].

**Fig. 1:**
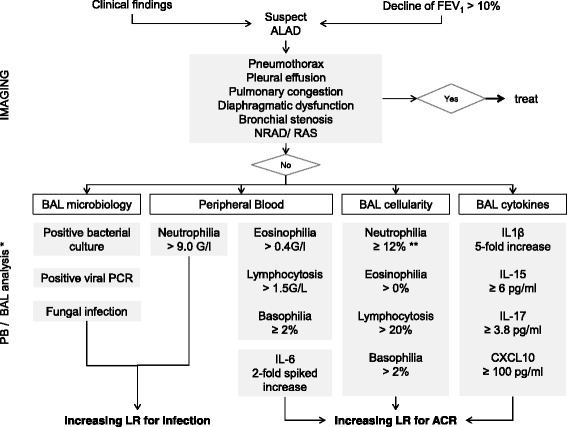
Proposal of an integrative algorithm. This descriptive algorithm is designed to estimate probabilities for ACR or other causes of ALAD in lung transplant recipients presenting with clinical findings or a drop in lung function (FEV_1_ > 10%). The integration of microbiology data, differential cytology [11] and cytokine expression levels from peripheral blood and BAL samples might assist in decision-making to increase or decrease the likelihood for ACR in the context of the clinical presentation. In the absence of standardized BAL techniques and detection methods, BAL cytokine expression levels and cut-offs have to interpreted wth caution and should be confirmed in larger studies. Since results from studies with very different designs have been included, direct translation in a clinical setting is not feasible and the use of this algorithm does not obviate the need for biopsy to confirm or exclude histology-proven ACR. * Numbers vary between different studies. ** Absence of microbiological evidence for infection

Table 3 and Table 4 list several cytokines that are present in BAL and PB samples. Moreover, anticipated changes during ACR and both sensitivity and specificity for the diagnosis of ACR are reported when available. Figure 1 shows a proposal for an integrative algorithm with clinical data, radiographic findings, BAL microbiology studies, BAL differential cytology and selected cytokines obtained from BAL and PB samples. Data concerning differential cytology are based on a previous review article in this journal [11]. Specific constellations in the levels of these factors might increase the suspicion for ACR. However, standardization of BAL techniques and detection methods are needed. Appropriate cut-off levels can then be determined for selected parameters to diagnose ACR based on this algorithmic approach. Cytokines and biomarkers involved are discussed below.

**Table 3:** Observed expression of cytokines in BAL samples during ACR

Cytokine	Expression in BAL	Reference	Measure	Sensitivity PPV	Specificity NPV
IFN- γ	↑	[55]	mRNA; during early ACR only		
	↑	[40, 56]	mRNA	77.7% 73.6%	85.7% 88.2%
	↑	[71]	Total RNA		
	0	[41]	mRNA		
	-	[72]	mRNA		
TNF- α	↑	[38, 69, 74]	mRNA (experimental)		
	↑0	[31]	Protein		
	0	[41]	mRNA		
	↓	[96]	mRNA expressed by AM		
	↑	[43]	mRNA expressed by AM		
	0	[97]	mRNA expressed by mononuclear cells		
TGF-β	-	[71]	mRNA		
	↓…↑		mRNA expressed by AM; dramatic increase 15 days after onset of ACR		
IL-1	↑	[31]	Protein		
	↑	[32]	mRNA expressed by AM		
IL-2	0	[55, 56]	mRNA	44.4% 40.0%	63.6% 67.7%
	-	[32]	mRNA expressed by AM		
IL-4	↑	[16]	mRNA		
	↑	[35]	mRNA expressed by CD8+ T cells		
	0	[34]	mRNA expressed by T cells		
	-	[32]	mRNA expressed by AM		
IL-5	0	[98]	Protein		
IL-6	↑	[38, 99]	mRNA (experimental)		
	↑	[16]	mRNA		
	↑	[40]	Protein		
	↑0	[31]	Protein		
	0	[41]	mRNA		
	0	[42]	Protein		
	↑	[32]	mRNA expressed by AM		
	↑0	[43, 44]	mRNA expressed by AM		
IL-8	↑0	[31, 50]	Protein		
	0	[51]	Protein and mRNA		
	0	[42]	Protein		
IL-10	↑	[31]	Protein		
	0	[55, 56]	mRNA	88.8% 41.0%	32.3% 84.6%
IL-15	↑	[32, 61]	mRNA expressed by AM		
	↑	[62]	Blocked IL-2 receptor		
	0	[41]	mRNA		
IL-16	(↓)	[100]	Protein		
	(↑)	[41]	mRNA		
IL-17	↑	[51, 66]	Protein		
	↑	[51]	mRNA		
	0	[41]	mRNA		
IL-18	0	[101]	Protein		
CXCL10	↑	[39, 41]	mRNA		
	↑	[80]	Protein		

0 Unchanged

↑ Increased

↓ Decreased

↑0 Increased without statistical significance

() Confounded effect

- Not detected

**Table 4:** Observed expression of cytokines in plasma samples during ACR

Cytokine	Expression in serum	Reference	Remark
IL-6	↑	[45]	Spiked elevation
	↑0	[46]	

↑ Increased

↑0 Increased without statistical significance

### IL-1

Interleukin-1 (IL-1) is a multifunctional pro-inflammatory cytokine, affecting nearly every cell type [27]. Two genes for IL-1 exist: IL-1α and IL-1β. In the context of transplantation, IL-1β has been associated with graft-versus-host disease (GVHD), ischemia-reperfusion-injury and the development of bronchiolitis obliterans (BO) [28–30].

Clinical studies observed an increase of IL-1 during periods of ACR. As such, IL-1β was significantly higher in patients with ACR compared to patients without rejection in a prospective study [31]. Moreover, the level of IL-1β was significantly raised during episodes of rejection compared to periods before rejection underlining the potential role of IL-1 as discriminator between rejection and infection. Another study found that BAL alveolar macrophages (AM) of patients with ACR expressed significantly more IL-1α and IL-1β compared to lung transplant recipients without rejection [32].

**Table:** 

IL-1 is elevated during ACR and might be a useful marker to distinguish between allograft rejection and allograft infection.	

### IL-4

IL-4 is an anti-inflammatory cytokine released by type 2 helper T cells (Th2 cells), basophils and mast cells [33]. While the effects of IL-4 in Th2 cells has been extensively studied, the response to IL-4 in other T cell types appear to be equally important [33].

Results on IL-4 in clinical studies are inconclusive. IL-4 could not be detected in AM from BAL samples in patients with rejection, infection and absence of both conditions [32]. In another study, the relative amount of T cells expressing IL-4 did not change between stable and rejecting groups [34].

Whitehead and colleagues identified IL-4 as a potentially useful marker for ACR [16]. When studying cytokines in BAL and PB samples from 31 lung transplant recipients, the authors detected IL-4 messenger RNA (mRNA) in less than 20% of all samples. Interestingly, IL-4 was not found in samples obtained from patients with infection. The greatest difference was observed when samples of patients with AR were compared to patients without AR. In addition, cytokine gene expression levels varied considerably between PB and BAL samples. This observation was confirmed in another study, where T cell cytokine production in BAL and PB was compared in stable lung transplant recipients and healthy volunteers [35]. In both groups, T cells (CD4+ and CD8+) produced significantly more IL-4 in BAL compared with PB. Furthermore, CD8+ T cells in BAL expressed significantly more IL-4 in transplant recipients than in healthy controls.

**Table:** 

Expression of IL-4 during ACR is unclear. IL-4 might not be found in lung transplant recipients with infection.	

### IL-6

IL-6 is one of the major pro-inflammatory cytokines that mediates hepatic acute-phase response [36]. In the lungs, IL-6 has been identified as a key player in vascular remodelling and pulmonary hypertension (reviewed in [37]).

The pro-inflammatory role of IL-6 in ACR has been investigated in both experimental and clinical studies. Rolfe and colleagues observed a bimodal pattern of IL-6 production in BAL fluid in an experimental in vivo model [38]. The investigators transplanted incompatible left lung allografts from Brown Norway rats into Lewis rats. IL-6 mRNA levels were increased in BAL fluid of the transplanted lung on day one and were decreased by day four before increasing again on day six compared to the native lung. The observed pattern coincided with histopathologic changes consistent with ACR.

IL-6 tends to be increased in BAL samples during ACR. As such, IL-6 was elevated during ACR in a recent prospective study [31]. However, this increase was not significant and it is of note that diagnosis of ACR was not based on TBB but on clinical and radiological criteria. IL-6 was also increased in BAL samples during ACR in another clinical study [16]. IL-6 and IL-4 increases were found more often in patients with ACR than any other cytokines changes. Similarly, IL-6 protein was higher in lung transplant recipients with histopathogical proven ACR compared with infection and levels of IL-6 were significantly increased during moderate or severe ACR [39]. In another study, IL-6 mRNA in BAL was highest in patients with corticosteroid-refractory histologic ACR [40].

Conversely, other studies found no significant association between IL-6 and ACR [41, 42].

Various researchers have investigated the expression of IL-6 by AM. Production of IL-6 by AM in BAL fluid was significantly increased during ACR compared to stable control patients [43, 44]. When BAL was repeated 15 days later, values had decreased, reaching similar levels as in stable control patients [43]. Furthermore, cytokine production was higher during symptomatic rejection episodes compared to silent rejection episodes and in ISHLT Grade II rejection compared to Grade I rejection. However, both differences were not significant. Similarly, in another study, AM secreted significantly more IL-6 during ACR than in patients without rejection or infection [32].

Monitoring IL-6 in serum after lung transplantation might be beneficial to detect ACR. However, level of evidence is low. Yoshida and colleagues found that a spiked elevation of IL-6 might predict the presence of ACR [45]. The investigators divided 17 patients into four groups according to the pattern of IL-6 elevation. 13 out of 19 spikes in the group with sharp raises of serum IL-6 levels were associated with histologically or clinically confirmed ACR. Conversely, continuously high levels of IL-6 were characteristic for infection. In another study, IL-6 was increased in serum during ACR, though this was not significant [46]. Interestingly, no association was found between the levels of IL-6 in serum and BAL.

**Table:** 

Increased IL-6 has been associated with ACR. Monitoring serum levels of IL-6 after lung transplantation might help to detect ACR. However, current level of evidence is low.	

### IL-8

IL-8 is a major chemotactic factor for neutrophils secreted by macrophages and respiratory epithelial cells [47]. In the lung, IL-8 is associated with lymphocytic airway inflammation and neutrophilic reversible allograft dysfunction (NRAD) [48, 49].

Some clinical studies have identified an increase of IL-8 in BAL fluid during ACR, yet this was not significant [31, 50]. Levels of IL-8 did not correlate with severity of ACR [50].

Other investigators did not notice any significant changes between nine rejecting patients and 17 non-rejecting controls as far as levels of IL-8 in BAL were concerned [51]. In a case–control study no significant difference was detected between IL-8 levels in BAL fluid of patients with ACR and stable controls [42]. While IL-8 has been clearly linked with CLAD, its role in ACR has yet to be elucidated.

**Table:** 

IL-8 has been associated with the development of BO, but a defined role of IL-8 in ACR has yet to be elucidated.	

### IL-10

IL-10 is an anti-inflammatory cytokine secreted by Th 2 cells and regulatory T cells. It attenuates inflammatory response by inhibition of T cells, monocytes and macrophages [52]. Several experimental in vivo studies showed that administration of IL-10 before transplantation improved graft acceptance and survival [53, 54]. Importantly, this effect was not observed when IL-10 was given post transplantation.

Clinical data on IL-10 in BAL are conflicting. IL-10 mRNA was found in most BAL samples of patients who had undergone lung transplantation, irrespective of whether or not ACR was diagnosed [55]. Similarly, levels of IL-10 mRNA did not correlate with ACR in another study, as this cytokine was present in most BAL samples regardless of whether or not ACR was present [56]. In contrast, Patella and colleagues detected an increase of IL-10 levels in BAL fluid during ACR compared to infection and baseline [31]. Interestingly, levels of IL-10 showed opposite trends during infection and ACR. While levels of IL-10 increased during ACR, the expression of IL-10 was low during bacterial and CMV infection.

IL-10 has been attributed a protective role against allograft rejection, as highlighted by lung biopsies from clinical and experimental studies [57, 58]. Zheng and co-workers observed that patients with an increased IL-10 production genotype were less likely to have two or more consecutive biopsy specimens of ≥ ISHLT Grade A2 rejection despite anti-rejection treatment (steroid bolus, steroid taper or anti-T cell globulin) during the first year post transplantation compared with intermediate or decreased IL-10 production genotypes [57]. The authors suggested that future genetic testing might help to identify patients at risk for multiple episodes of ACR, which might help to tailor therapeutic regimens. The balance between graft destruction and regulation determines graft survival [59]. Regulatory immune cells in a recipient can shift the balance towards graft survival. Interestingly, one of the common features of many regulatory cells is their ability to secrete IL-10 [59]. The observed increase of IL-10 during ACR might thus reflect the attempt to create an anti-rejection microenvironment.

Using a rat model, Oishi and co-workers assessed the effect of ex vivo lipid-mediated transbronchial human IL-10 gene transfer [58] and examined the intra-graft cytokine profile on day three and six after single-lung transplantation between highly histo-incompatible rats. The stage of ACR and different pathologic markers for inflammation were lower in the group receiving intra-bronchial plasmids encoding IL-10 compared to the control group receiving mock plasmids. Furthermore, ex vivo lipid-mediated transbronchial human IL-10 gene transfer was associated with lower levels of IL-2 and TNF-α mRNA on day three post transplantation [58].

**Table:** 

Increased IL-10 has been attributed a protective role against inflammation. BAL levels show no clear consistent pattern. However, IL-10 levels increased in ACR and decreased in infection in one study.	

### IL-15

IL-15 is a pro-inflammatory cytokine secreted by multiple tissues and cell types, among which macrophages play a principal role [60]. IL-15 is similar to IL-2 in its structure and biological function. It is a potent chemo-attractant factor for T cells and is involved in B cell maturation and NK cell cytotoxicity [60].

The few results on levels of IL-15 in BAL are promising in the context of ACR. According to Rizzo and colleagues, IL-15 expression by BAL-derived AM was increased in five out of nine patients experiencing ACR compared to patients without rejection and infection [32]. In the other four patients with ACR no IL-15 was detected. In the same study, the authors prospectively assessed cytokines in three patients during and after treatment of ACR, noticing that levels of IL-15 decreased with resolution of ACR. Shi and co-workers found a similar trend: IL-15 mRNA expressed by BAL AM was significantly higher in 15 patients with ACR than in 19 stable controls [61]. In another study, IL-15 protein was elevated in BAL during ACR when the IL-2 receptor was blocked [62]. This study was conducted based on the assumption that IL-2 and IL-15 share similar functions and on the observation that blocked IL-2 receptors do not completely avert ACR. Three interesting observations were made: The average IL-15 protein level was increased in acutely rejecting patients compared to stable controls, IL-15 was distributed in a bimodal fashion in patients undergoing ACR, and levels of IL-15 did not correlate with infection. In contrast to the trends mentioned above, Husain and colleagues did not find any significant association between IL-15 and ACR [41].

**Table:** 

Elevated IL-15 expressed by AM has been associated with ACR. When the alpha chain subunit of the IL-2 receptor was blocked IL-15 was increased.	

### IL-17

IL-17 is a pro-inflammatory cytokine, which is primarily secreted by CD4+ T cells [63]. IL-17 has been reported to play an important role in sustained mobilisation of neutrophils via induction of IL-8 [64]. In this context, it has been linked with chronic inflammatory lung diseases, although its causative role is unclear [65].

Various studies have suggested a role for IL-17 in acute and chronic rejection after lung transplantation [51, 66, 67]. Vanaudenaerde and co-workers found an increase of IL-17 in BAL fluid during ACR on day 28 after lung transplantation [51]. IL-17 mRNA was raised significantly compared to stable controls. IL-17 protein was also increased, however, without reaching significance. At the same time, raised IL-17 protein levels were associated with higher lymphocytes in BAL fluid. Interestingly, IL-17 protein correlated with severity of rejection. When BAL fluid was analysed on day 90 after transplantation, IL-17 was no longer elevated compared with stable control patients. It remains unclear whether this finding is due to immunosuppressive therapy or whether it represents the natural course after ACR. Conversely, Husain and colleagues did not find any association between IL-17 levels and ACR [41].

**Table:** 

IL-17 has been associated with mobilisation of neutrophils and chronic inflammatory lung disease. Levels of IL-17 were increased in BAL during ACR.	

### IFN-γ

Interferon gamma (IFN-γ) is a pro-inflammatory cytokine secreted by type 1 helper T cells (Th1 cells) and natural killer cells (NK cells) in response to IL-12 from macrophages. It promotes macrophages to degrade phagocytosed pathogens and inhibits differentiation of Th2 cells [68]. It is further involved in NK cell activity and B cell regulation [68].

IFN-γ was found to be raised in animal models during ACR [69, 70]. In a rat model, Brown Norway rat lungs were depleted of AM before transplantation into Lewis rats [70]. While production of IFN-γ was significantly reduced in BAL fluid after transplantation, the development of ACR could not be prevented.

Results obtained in clinical studies remain inconclusive. Expression of IFN-γ mRNA in BAL fluid has been associated with ACR in several studies [55, 56]. In a prospective study mRNA expression of IFN-γ in BAL fluid was significantly higher during early ACR (defined by occurrence within three months following transplantation) but not during late ACR [55]. In another study, the presence of IFN-γ in BAL fluid predicted ACR with a specificity of 85.7% and a sensitivity of 77.7% (positive predictive value (PPV) 73.6%, negative predictive value (NPV) 88.2%) [56]. Moreover, when IFN-γ gene was expressed during ACR, FEV_1_ declined more than in the absence of IFN-γ expression. In another study, BAL cell-derived IFN-γ mRNA was highest in patients with refractory ACR that did not respond to high-dose intravenous corticosteroid therapy compared to non-rejecting or steroid-responsive control-groups [40]. After treatment with aerosolized cyclosporine IFN- γ decreased significantly [40]. By performing gene expression microarrays, IFN-γ was found to be elevated in all seven samples with ACR whereas it was decreased or absent in the other 27 non-rejection samples [71].

Other studies found no significant association between IFN-γ and ACR or failed to detect IFN-γ mRNA in BAL samples from patients with ACR [41, 72].

**Table:** 

IFN-γ has been found at increased levels in BAL samples during ACR.	

### TNF-α

Tumor necrosis factor alpha (TNF-α) is a potent pro-inflammatory cytokine secreted by macrophages, T cells and NK cells that directs homeostatic and pathogenic functions [73].

TNF-α was increased in animal models during ACR [38, 69, 74]. More specifically, TNF-α was raised at cellular and molecular levels in BAL samples of a rat model [74]. Production of TNF-α mRNA was highest during severe ACR. Moreover, the extent of the bioactivity of TNF-α was shown to correlate with the histopathological grade of rejection. Interestingly, animals receiving intravenous injections with TNF-α antisera before transplantation showed markedly attenuated ACR compared with rats receiving control sera [74]. In another experimental study, donor rat lung allografts (Brown Norway) that had been depleted of AM before transplantation into recipients of a different strand (Lewis) showed significantly lower levels of TNF-α compared with untreated donor lungs in BAL fluid [70]. While this depletion affected the production of several IgG subclasses, it did not prevent the development of ACR.

Data obtained by clinical studies remain unclear. Husain and colleagues found no significant association between the expression TNF-α in BAL fluid and ACR in a prospective study [41]. The authors explained this by the low levels of TNF-α in the BAL samples, probably due to the lower number of AM detected.

In a previous study that analysed levels of TNF-α RNA in 26 BAL and TBB samples, TNF-α was expressed primarily in inflammation due to unspecified causes and less in ACR [72]. Conversely, Patella and colleagues reported an increased expression of TNF-α during ACR, however this finding was not significant [31].

Inconsistent results between studies analysing the expression of TNF-α by AM in BAL might be explained by different methods for culturing macrophages and lack of standardization. However, studies on biomaterials did not show relevant differences in cytokine expression from macrophages by different surface chemistries used to culture these cells [75]. TNF-α was significantly increased in the culture of BAL AM during ACR compared to clinically stable periods [43]. Also, cytokine levels were higher during symptomatic periods of ACR and in ISHLT Grade II rejection compared to asymptomatic periods and grade I rejection, respectively. In BAL samples analysed 15 days after ACR, TNF-α levels had decreased and were comparable to the levels of patients without rejection. In another study AM expressed TNF-α in five out of nine patients during ACR, while TNF-α could not be detected in the remaining four patients [32]. Another study found contrasting results; TNF-α secretion was significantly lower during ACR compared to levels in patients with no complication or infection after transplantation [76].

**Table:** 

TNF-α levels were increased in BAL samples of experimental models mimicking ACR. Data in human lung transplant recipients are less clear.	

### TGF-β

Transforming growth factor beta (TGF-β) is an anti-inflammatory cytokine also known for its profibrotic role [77]. Dysfunction of TGF-β has been observed in autoimmune diseases or in in conditions with impaired immune surveillance [78]. Among the three isoforms TGF-β1, −β2 and -β3 found in mammals, TGF-β1 predominates with strong immune-regulatory properties [79].

Increased TGF-β has been linked to the late phase of ACR and tissue repair during an inflammatory reaction. Magnan and colleagues examined the secretion of IL-6 and TGF-β by AM during ACR and other complications after lung transplantation [44]. Interestingly, the secretion of IL-6 and TGF-β by AM showed an opposite trend with time: IL-6 was elevated at the onset of ACR and returned to control values 15 days after diagnosis of AR and treatment. Conversely, the average concentration of TGF-β in AM supernatants was normal at the beginning and increased dramatically by 15 days after onset of ACR. This observation mirrors the inflammatory reaction in lung tissue, characterised by a phase of lung injury, followed by a phase of tissue repair.

In another study that used gene expression microarrays to identify new biomarkers for ACR, TGF-β was not found to be up-regulated [71]. However, many downstream mediators were increased, including the TGF-β receptor.

**Table:** 

TGF-β has been associated with the phase of tissue repair and was significantly increased 15 days following ACR and treatment in one study.	

### CXCL10

CXCL10 is a chemokine induced by IFN-γ. It has been shown to promote the directional migration of activated T cells during inflammatory immune responses [80]. CXCL10 has been strongly associated with graft survival after transplantation of various organs, including kidney, heart and GVHD [81].

Increased CXCL10 has been associated with ACR in clinical studies [39, 41]. CXCL-10 was higher in patients with ACR than in patients with infection [41]. This finding might help differentiate ACR from infection in transplant recipients with deterioration of graft function. Agostini and colleagues identified a correlation between high levels of CXCL10 protein in BAL and early ACR (ISHLT Grade A1-A3) and late rejection [80]. The authors furthermore emphasised that CXCL10 and its receptor CXCR3 might provide suitable molecular targets to decrease T cell recruitment and inhibit T cell-specific inflammatory processes in the lung. In another study, cumulative exposure of lung allograft recipients to CXCL10 correlated with a significant risk of graft failure [82]. CXCL10 was also elevated before the onset of BO. CXCL-10 might not be specific for rejection processes, as it has also been detected in lungs with sarcoid granulomatous reactions [83].

**Table:** 

Elevated levels of CXCL10 have been found in several clinical studies during ACR. The use of CXCL10 as diagnostic marker (or its receptor CXCR3 as therapeutic target) might be limited by the lack of specificity for allograft rejection.	

### Summary

Studies about the level of cytokines in BAL during ACR show inconsistent results. This is probably due to discrepancies between BAL techniques, laboratory methods and the complexity of the cytokine network. Nevertheless, changes in cytokine expression have been observed in BAL samples during ACR, among which an increase in IL-1, IL-6, IL-15, IL-17 and CXCL10 seems most consistent.

Few studies assessed cytokine levels in serum. A sharp increase in serum IL-6 seems to be associated with ACR. The scarcity of serum data is likely due to the on-going controversy whether pathological changes in a solid organ of the body are accurately reflected by blood mediators assessed in the systemic circulation [14, 24].

### Limitations

Data from the studies included in this review should be interpreted with caution. Seriously diverging methodologies impair direct comparison between studies and might explain discrepancies in the cytokine profiles between various laboratories investigating the same molecules [11, 12].

At present, measurements of cytokine levels in BAL samples are not standard of care in lung transplant patients. Different BAL techniques and several sampling issues limit the direct comparability of BAL results [84, 85]. More specifically, the composition of BAL fluid is influenced by dilution factors related to the BAL technique, alveolar permeability at the time of sampling and contamination by bronchial fluid [85, 86]. Moreover, pattern and volume of instilled fluid are important issues and might alter cytokine levels. The ISHLT has recently established a working group on BAL standardization to address these issues in the context of lung transplantation [Tereza Martinu, Toronto, Canada, personal communication].

Furthermore, cytokine detection methods lack standardization and reproducibility [12, 87]. ELISAs are currently the preferred method to measure cytokine levels as outlined above [12]. However, sensitivity of different ELISA kits and reproducibility within the same kit vary substantially, resulting in a wide variation of values [88, 89].

Few of the cytokines described in this article have been investigated in a prospective validation study, moreover, data on the levels of healthy controls are rare and no threshold levels have been set for ACR after lung transplantation. Consequently, such cut-off levels should be defined and sensitivity, specificity and predictive values should then be established in a multi-centre cohort study.

All these factors limit comparability, conclusions and direct translation of these findings into a clinical setting. For future research it is imperative that the methods to identify potential biomarkers of ACR are standardised and validated. Potential molecules should then be assessed prospectively in multi-centre studies with large cohorts, using consistent, validated methods.

Standardized retrieval and detection methods would ultimately allow reliable data analysis and integration. Considering the pleiotropic nature and redundancy of cytokines, creating a composite score that integrates several cytokines will enhance the diagnostic value of the BAL cytokine profile. Cytokine patterns could further be merged with changes in cellularity to increase sensitivity and specificity, as seen in the context of CLAD [42]. However, data complexity will make translation into a clinical setting more challenging.

## Conclusions

Changes in cytokines of BAL and plasma samples during ACR in the lung have been identified. Whereas an increase in IL-1, IL-6, IL-15, IL-17 and CXCL10 in BAL fluid might warrant suspicion for ACR, levels of IL-8 and IL-18 do not appear to be suitable as diagnostic markers during ACR. Data from blood analysis remain weak with very few studies assessing cytokine changes in PB during ACR. A spiked elevation of IL-6 has been associated with ACR. Overall, no strong associations were found that would allow relying exclusively on single cytokine profile changes in BAL or PB samples for diagnosis of ACR. Combinations of cytokine alterations may eventually contribute to a composite score drawing from more than one marker setting (BAL and PB cytokines and cytology), which may increase diagnostic accuracy and clinical use.

The proposed algorithm in Fig. 1 integrates different clinical data to increase the likelihood for ACR and warrant bronchoscopy to obtain biopsies. It has to be reminded, however, that even TBB and BAL results have limitations for detecting rejection and infection and other causes of allograft dysfunction have always to be considered.

To strengthen the reproducibility of this data it might be beneficial to routinely measure protein levels of IL-1, IL-6, IL-15, IL-17 and CXCL10 when performing BAL analyses during bronchoscopy of asymptomatic patients as well as in patients with suspicion of ACR and other pathologies of the lung and airways. Blood samples could be routinely screened for levels of IL-6 protein. This will allow defining a normal cytokine profile in BAL and PB as well as cut-off values for ACR, as shown in the context of BAL cytology [90].

Advancing research on non-invasive biomarkers may eventually improve patient care. Using less-invasive assays will help to i) detect ACR at an earlier stage, ii) determine subclinical rejection, iii) develop drugs targeting specific molecular structures, iv) identify allograft recipients who benefit from reduction or modification of immunosuppressive drugs and v) recognize patients at risk for CLAD not identified by TBB [8].

So far cytokine profile analysis is not part of the routine diagnostic workup for suspected ACR. When used within the clinical context, cytokine and cytology profiles of BAL and serum samples might be useful to assist in decision-making and alter the likelihood for the presence or absence of ACR. Overall, BAL and serum samples are no substitutes for TBBs in the evaluation of lung function decline in lung transplant recipients, but may increase the diagnostic yield of bronchoscopy for detection of ACR**.**

## Abbreviations

ACR: acute cellular rejection
AM: alveolar macrophages
BAL: bronchoalveolar lavage
BO: bronchiolitis obliterans
CLAD: chronic lung allograft dysfunction
CSF: colony-stimulating factor
ELISA: enzyme-linked immunosorbent assay
FCM: flow cytometry
GVHD: graft-versus-host disease
IFN: interferon
IFN-γ: interferon gamma
IL: interleukin
mRNA: messenger RNA
NK cells: natural killer cells
NPV: negative predictive value
NRAD: neutrophilic reversible allograft dysfunction
PB: peripheral blood
PCR: polymerase chain reaction
PPV: positive predictive value
RAS: restrictive allograft syndrome
RT-PCR: reverse transcription polymerase chain reaction
TBB: transbronchial biopsy
TGF: transforming growth factor
TGF-β: transforming growth factor beta
Th1 cell: type 1 helper T cell
Th2 cell: type 2 helper T cell
TNF: tumor necrosis factor
TNF-α: tumor necrosis factor alpha
